# Expectations of emergency patients regarding triage system knowledge upon arrival: an interpretive study

**DOI:** 10.1007/s11845-024-03706-5

**Published:** 2024-05-13

**Authors:** Mohammad Minwer Alnaeem, Salam Salam Banihani, Asma Islaih, Ahmad R. Al-Qudimat

**Affiliations:** 1 School of Nursing, Al-Zaytoonah University of Jordan, Amman, Jordan; 2 School of Nursing, Irbid National University, Irbid, Jordan; 3 School of Nursing, Al-Zaytoonah University of Jordan, Amman, Jordan; 4 Surgical Research Section, Department of Surgery, Hamad Medical Corporation, Doha, Qatar; 5 Department of Public Health, College of Health Sciences, QU-Health, Qatar University, Doha, Qatar

**Keywords:** Arrival, Emergency department, Expectation, Knowledge, Triage system

## Abstract

**Background:**

One of the most important aspects of healthcare knowledge is having a thorough understanding of the triage system which is used in emergency departments. This study aims to assess the level of awareness of Jordanian patients who visit the ED about the triage procedure.

**Methods:**

A descriptive, cross-sectional design was utilized in the emergency department at the biggest public hospital in Jordan. A convenience sample of a self-administrated questionnaire utilizing a Discounted Cash Flow Interview (DCF) survey was filled out.

**Results:**

A total of 726 participants were recruited with a response rate of 90.8%. The mean age of the participants was *M* = 38.1 (SD = 12.9), and the age of the participants varied from 18 to 89 years. More than half of the participants were male (*n* = 383, 52.8%) and married (*n* = 425, 58.5%). A significant relationship between the overall perception of knowing what a teaching hospital is and patients’ educational level (*X*^2^ = 11.9, *P* < 0.003), current job (*X*^2^ = 25.2, *P* < 0.001), nationality (*X*^2^ = 7.20, *P* < 0.007), and family income (*X*^2^ = 15.9, *P* < 0.001).

**Conclusion:**

More investigation is required to determine the causes of the low knowledge of the triage system. The study suggests increasing staffing levels, giving nursing staff ongoing education and training, and integrating technology and automation to reduce the load of patient care.

## Introduction

One of the most important aspects of healthcare knowledge is having a thorough understanding of the triage system which is used in emergency departments [1, 2]. When a patient seeks emergency care, the triage system is the first place they visit [3]. Trained medical personnel, usually nurses, are in charge of determining the kind and severity of the patient’s ailment [4]. This system, informed by established international frameworks such as the Canadian model, involves a meticulous classification of cases into distinct urgency levels, ranging from immediate to less urgent (Costa, Nicolaidis, Gonçalves, Souza, & Blatt, [5]).

In the dynamic environment of emergency care, the triage system is instrumental in facilitating a systematic and prioritized approach to patient management [6]. Healthcare providers in emergency departments (EDs) rely on this system to efficiently allocate resources promptly with appropriate interventions to be administered to those with critical needs [7]. The procedure calls for both clinical and visual assessments, which require a sophisticated comprehension of a range of medical issues and the quick decision-making skills necessary to directly affect patient outcomes [8]. Despite the critical role of the triage system in optimizing emergency healthcare delivery, challenges arise in its execution [9]. Managing both urgent and non-urgent situations in the same setting is one of these difficulties, which could result in decreased operational effectiveness and longer patient wait times [10].

One major problem in healthcare settings is that patients are not aware of the triage mechanism in place in emergency departments. Patients’ expectations about the severity of their ailment are sometimes misaligned due to the lack of knowledge about the triage procedure, which causes inappropriate or delayed care [11–13]. According to a study performed by AlShatarat et al. [14], it was reported that lack of awareness can contribute to overcrowding in EDs, hampering the timely management of critical cases and potentially increasing patient morbidity and mortality rates.

In addition, patients may not actively engage in the triage process if they are not aware of its guiding principles, which makes it difficult for medical staff to effectively distribute resources and prioritize care according to the severity of each case. This lack of understanding of the triage process highlights the crucial role of educational programs to make sure that they understand the vital role of the triage system in reducing wait times and improving patient outcomes in EDs [15].

It is essential to educate people about the triage procedure in Jordan, where emergency departments deal with large patient loads [14]. To improve patient care and raise knowledge of the triage system, it can be beneficial to promote patient awareness by improving resource allocation and decreasing wait times (Al-Kalaldeh, Al-Bdour, & Shosha, [16]). In Jordan, patients’ awareness of the triage system is still lacking. Nevertheless, educating the public about the ED’s workflow and patients’ rights and responsibilities is crucial to the department’s operation as well as to staff productivity [17]. Thus, in-depth comprehension of the triage system is indispensable for healthcare professionals operating in emergency settings, contributing significantly to the enhancement of patient outcomes and overall system efficacy; the purpose of this study was to assess the level of awareness of Jordanian patients who visit ED about the triage procedure.

## Methods

### Design

A descriptive, cross-sectional design was utilized.

### Setting and population

The study was conducted in the emergency department at various biggest public hospitals located in the capital of Jordan (Amman) which provides care for many patients with different conditions. These hospitals are administratively affiliated with the Ministry of Health and are accredited as educational hospitals by the Jordanian Medical Council and the Arab Council for Medical Specializations. These hospitals serve about 4.5 million people (as per the 2021 census), and they have different EDs which provide care for 45% of the Jordanian population with different medical conditions which could reach over 12,000 patients daily (Ministry of Health, 2023). Convenience sampling was used to recruit the participants. The minimum sample size was 619 participants based on the G* Power software calculation (power of 0.95, *α* = 0.05, medium effect size 0.2) [18].

### Study measurements

A self-reported questionnaire that consists of two tools was used. The first part is composed of a socio-demographic data sheet that includes age, sex, educational level, current job, marital status, residency place, nationality, and monthly income in Jordanian dinar. The second part is concerned with the Discounted Cash Flow Interview (DCF) survey that was developed by Seibert et al. [19] in the USA to assess the ED visitor’s knowledge about the ED process and hospital function. An Arabic version of Alhabdan et al. [20] was used in this study. This tool was used to measure the patient’s awareness of the quality of nursing care in hospitals. The awareness section included four domains. However, two domains were used to accomplish the purpose of this study, namely, knowledge of the emergency triage system (five items,two open-ended and three multiple-choice questions) and the ED visitor’s expectations that have questions with varied answers, as they contain open-ended answers and multiple-choice questions. This tool was valid and reliable, with a Cronbach’s alpha of 0.77–0.83 [19, 20].

### Ethical consideration

The study was approved by Al-Zaytoonah University of Jordan (No. 2024–2023/133/03) and the selected hospital (No. MOH/REC/2023/480). Informed consent was included with full details of the purpose, benefits, risks, and time required to fill out the questionnaire. Data were kept secure and anonymized.

### Data collection procedure

After getting approval from the required parties, a meeting with the head nurse in the emergency department was held to explain the purpose of the study. An interview with patients took place in the triage room. The participants signed the consent form and completed the questionnaire. The researcher stored the completed questionnaires in a sealed envelope, ensuring that only the researcher had access to the data. The study was conducted from June to September 2023, with all envelopes stored in the researcher’s office.

### Data analysis

Statistical Package for Social Science version 28 was used to analyze the collected data. Descriptive and inferential statistics were used to describe the sample and assess patients’ awareness of the triage system at the ED in Jordan. The chi-square test was used to find out the relationship between two categorical variables. *P*-value was significant at < 0.05.

## Results

A total of 800 questionnaires were distributed, and 74 questionnaires were excluded (due to incomplete responses); thus, the final sample for analysis was 726 participants, achieving a response rate of 90.8%.

### Study participant’s characteristics

The mean age of the participants was *M* = 38.1 (SD = 12.9), and the age of the participants varied from 18 to 89 years. More than half of the participants were male (*n* = 383, 52.8%) and married (*n* = 425, 58.5%). More than half of the participants hold a diploma and higher degree of education (*n* = 399, 54.9%), while there were (*n* = 126, 17.4%) illiterate. The majority of participants (*n* = 649, 89.4%) were Jordanians and lived in the capital of Jordan, Amman (*n* = 430, 59.2%), as shown in Table 1.

**Table 1:** Characteristics of the sample (*n* = 726)

**Variable**	***n*(%)**
** Age** Mean **± **SD Median** (***P*_**50%**_**)** Minimum**–**maximum	38.1 ± 12.2 36 18–89
** Sex** Male Female	383 (52.8) 343 (47.2)
** Education level** Illiterate Completed high school Diploma or higher	126 (17.4) 201 (27.7) 399 (54.9)
**Current job** Do not work Healthcare work Governmental work Private work	287 (39.6) 147 (20.2) 139 (19.1) 153 (21.1)
**Marital status** Married Single Others	425 (58.5) 190 (26.2) 109 (15.3)
**Nationality** Jordanian Non-Jordanian	649 (89.4) 77 (10.6)
**Place of residence** In Amman Out Amman	430 (59.2) 296 (40.8)
**Monthly income (Jordanian dinars)** < 260 260 to 400 > 400	159 (21.9) 172 (23.7) 395 (54.4)

*SD* standard deviation, *n* number, *%* percentage, *M* mean

### Knowledge about the triage system

Regarding participants’ knowledge of the triage system, the majority of participants (*n* = 445, 61.3%) did not know what the triage system entailed. Over half of the participants (*n* = 424, 58.4%) are aware of what an educational hospital is and that they are currently receiving medical care services. Most patients (*n* = 434, 59.8%) know why some patients were taken to a room before others (even though they may not have waited a long time). The majority of participants think the triaging system is fair enough for all (*n* = 534, 73.6%). Nonetheless, 61.3% (*n* = 445) of the participants were ignorant of the meaning of triage, as shown in Table 2.

**Table 2:** Knowledge about the triage system (*n* = 726)

**Items**	**Yes**	**No**
	***n* (%)**	***n* (%)**
**Do you know what a teaching hospital is?**	424 (58.4)	302 (41.6)
**Do you know if this hospital is a teaching hospital?**	402 (55.4)	324 (44.6)
**Do you know why some patients are taken to a room before others even though they may not have waited as long?**	434 (59.8)	292 (40.2)
**Do you think this is fair?**	534 (73.6)	192 (26.4)
**Do you know what triage means?**	281 (38.7)	445 (61.3)

*n* number, *%* percentage

### Visitor’s expectations in emergency

In terms of ED visitor’s expectations, most of the participants did not want to know how long other patients waited (*n* = 446, 61.4%). However, many of the participants agreed to hear updates about possible delays (*n* = 435, 59.9%); of them, 67.9% (*n* = 493) wanted to know updates about delays every half an hour. The majority of people (*n* = 231, 31.8%) who preferred to hear from healthcare practitioners about any updates regarding delays were nurses. Further, the majority of participants want to know further information about how the ED works (*n* = 601, 82.8%), especially about hypertension, diabetes, or cancer (*n* = 565, 77.8%) and how to find a primary care provider (*n* = 569, 78.4%). For the overall perception, participants reported that being presented in a teaching hospital might affect the care provided for them as they believe that teaching hospitals have more emphasis on patient education (*n* = 426, 58.7%), as shown in Table 3.

**Table 3:** The ED visitor’s expectations (*n* = 726)

**Item**	***n*(%)**
**Do you want to know how long other patients have been waiting?** Yes No	280 (38.6) 446 (61.4)
**Do you want to hear updates about delays to be seen?** Yes No	435 (59.9) 291 (40.1)
**If the last question was yes (or go to the next question), how often?** Every 30 min Every hour Every 2 h Every 3 h It does not matter	493 (67.9) 105 (14.5) 29 (4.0) 13 (1.8) 86 (11.8)
**Who should do the updates?** A clerk A nurse A physician It does not matter	184 (25.3) 231 (31.8) 143 (19.7) 168 (23.1)
**Do you want more information about how the ED functions?** Yes No	601 (82.8) 125 (17.2)
**If the last question was yes (or go to the next question), how would you like the information?** Handouts A video playing in the waiting room A computer with an educational module on it Others	170 (23.4) 304 (41.9) 204 (28.1) 48 (6.60)
**Do you want to know why you have to wait (for example, shortage of beds or other critical patients)?** Yes No	587 (80.9) 139 (19.1)
**Periodic updates from ED staff about the delays** Not important Important	440 (60.6) 286 (39.4)
**General information about common illnesses like high blood pressure, diabetes, or cancer prevention** Not important Important	161 (22.2) 565 (77.8)
**Information about the health care system and how to find a primary care provider** Not important Important	157 (21.6) 569 (78.4)
**Information about triage and how the ED functions** Not important Important	158 (21.8) 568 (78.3)
**Information on medical conditions (stroke, heart attack)** Not important Important	152 (21.1) 574 (79.0)

*n* number, *%* percentage

The chi-square test was used to assess the relationship between participants’ knowledge about the triage system during the patient’s ED visit and demographical data which revealed a significant relationship between the overall perception of knowing what a teaching hospital is and patients’ educational level (*X*^2^ = 11.9, *P* < 0.003), current job (*X*^2^ = 25.2, *P* < 0.001), nationality (*X*^2^ = 7.20, *P* < 0.007), and family income (*X*^2^ = 15.9, *P* < 0.001). Knowing if this hospital is a teaching hospital was significantly associated with education level *X*^2^ = 16.5, *P* < 0.001), current job (*X*^2^ = 22.9, *P* < 0.001), nationality (*X*^2^ = 9.39, *P* < 0.002), and family income (*X*^2^ = 9.20, *P* < 0.010).

Furthermore, the participants’ education level (*X*^2^ = 17.9, *P* < 0.000), current job (*X*^2^ = 18.5, *P* < 0.000), and nationality (*X*^2^ = 10.3, *P* < 0.001) were substantially correlated with their ability to understand why certain patients are transported to a room before others even though they may not have waited as long as they were waiting. Furthermore, there was a strong correlation found between patients’ education level (*X*^2^ = 6.90, *P* < 0.032), present job (*X*^2^ = 13.4, *P* < 0.004), and nationality (*X*^2^ = 20.7, *P* < 0.001) and the belief that some patients are taken to a room before others. Knowing what triage means also had a significant relationship with participants’ education level (*X*^2^ = 15.6, *P* < 0.001), current job (*X*^2^ = 14.9, *P* < 0.002), marital status (*X*^2^ = 7.26, *P* < 0.027), and nationality (*X*^2^ = 7.15, *P* < 0.008). However, the other items of the knowledge about triage system variables were not significantly concerning their sociodemographic variables (*P* > 0.05), as shown in Table 4.

**Table 4:** Relationship between knowledge about triage system and sociodemographic variables

Items	Do you know what a teaching hospital is?		Do you know if this hospital is a teaching hospital?		Do you know why some patients are taken to a room before others even though they may not have waited as long?		Do you think this is fair?		Do you know what triage means?	
	***X***^**2**^	***P***	***X***^**2**^	***P***	***X***^**2**^	***P***	***X***^**2**^	***P***	***X***^**2**^	***P***
**Age**	1.40	.238	0.68	.408	.21	.647	1.98	.160	.74	.391
**Gender**	2.42	.120	1.07	.300	.38	.540	.21	.648	1.99	.158
**Education**	11.9	.003*	16.5	.001*	17.9	.001*	6.90	.032*	15.6	.000*
**Current job**	25.2	.001*	22.9	.001*	18.46	.001*	13.4	.004*	14.9	.002*
**Marital status**	.76	.685	0.19	.908	1.99	.369	2.98	.223	7.26	.027*
**Nationality**	7.20	.007*	9.39	.002*	10.3	.001*	20.7	.001*	7.15	.008*
**Place of residence**	.03	.863	1.51	.219	1.50	0.22	.541	.464	.75	.388
**Family income**	15.9	.001*	9.20	.010*	1.49	4.58	.10	.476	1.95	.377

*X*^*2*^ chi-square analysis

^*^Significant at < 0.05

Furthermore, the chi-square test was used to assess the relationship between sociodemographic data and ED visitors’ expectations. The results showed that there was a statistically significant relationship between participants who desired to know how long other patients have been waiting and their age (*X*^2^ = 4.02, *P* < 0.045), education level (*X*^2^ = 13.5, *P* < 0.001), and current job (*X*^2^ = 9.90, *P* < 0.019). A desire to hear updates about delays was statistically significantly correlated with a current job (*X*^2^ = 23.8, *P* < 0.000), while the time for hearing those updates is significantly correlated with the current job (*X*^2^ = 31.63, *P* < 0.000) and place of residence (*X*^2^ = 14.4, *P* < 0.002). The desire to know more information about how the ED functions were significantly related to gender (*X*^2^ = 27.1, *P* < 0.000) and residency place (*X*^*2*^ = 4.64, *P* < 0.031) is shown in Table 5.

**Table 5:** Relationship between ED visitor’s expectations and sociodemographic variables

Variable	Do you want to know how long other patients have been waiting?		Do you want to hear updates about delays to be seen?		Who should do the updates?		Do you want more information about how the ED functions?		Do you want to know why you have to wait (for example, shortage of beds or other critical patients)?	
	***X***^**2**^	***P***	***X***^**2**^	***P***	***X***^**2**^	***P***	***X***^**2**^	***P***	***X***^**2**^	***P***
**Age**	4.02	.045*	.14	.706	0.65	.886	2.98	.084	1.47	.226
**Gender**	.010	.965	1.66	.198	6.79	.079	4.64	.031*	.02	.899
**Education**	13.5	.001*	4.21	.122	.98	.986	1.34	.511	2.62	.270
**Current job**	9.90	.019*	23.8	.001*	31.6	.001*	3.58	.311	3.0	.392
**Marital status**	2.08	.354	5.77	.056	4.41	.621	1.77	.413	1.47	.480
**Nationality**	.45	.504	2.08	.149	1.63	.652	2.82	.093	.99	.318
**Residency place**	.02	.896	0.17	.684	14.4	.002*	27.1	.001*	2.56	.110
**Family income**	.22	.898	0.95	.623	6.43	.376	2.96	.228	0.67	.716

*X*^*2*^ chi-square analysis

^*^Significant at < 0.05

Furthermore, periodic updates from the ED staff about the delays were statistically related to participants’ age (*X*^2^ = 3.29, *P* < 0.049), current job (*X*^2^ = 28.4, *P* < 0.019), marital status (*X*^2^ = 8.93, *P* < 0.012), and place of residence (*X*^2^ = 9.94, *P* < 0.002). Also, getting general information about common illnesses such as high blood pressure, diabetes, or cancer prevention was related to participants’ residency place (*X*^2^ = 5.90, *P* < 0.015). Receiving general information about the healthcare system and how to find a primary care provider was associated with participants’ educational level (*X*^2^ = 19.15, *P* < 0.010). Acquiring general information about triage and ED functions was related to participants’ gender (*X*^2^ = 3.48, *P* < 0.042). The medical conditions (e.g., stroke, heart attack) were significantly related to their education level (*X*^2^ = 5.81, *P* < 0.045) and nationality (*X*^2^ = 8.57, *P* < 0.003). However, the remaining items were not significantly associated with visitors’ expectations about ED variables (*P* > 0.05), as shown in Table 6.

**Table 6:** Relationship between patients’ sociodemographic variables and the ED visitor’s Expectations (*n* = 726)

Variable	Periodic updates from ED staff about the delays		General information about common illnesses like high blood pressure, diabetes, or cancer prevention		Information about the health care system and how to find a primary care provider		Information about triage and how the ED functions		Information on medical conditions (stroke, heart attack)	
	***X***^**2**^	***P***	***X***^**2**^	***P***	***X***^**2**^	***P***	***X***^**2**^	***P***	***X***^**2**^	***P***
**Age**	3.29	.049*	.34	.557	.36	.547	.02	.882	.12	.728
**Gender**	1.83	.177	2.56	.110	.26	.610	3.48	.042*	.77	.379
**Education**	.54	.762	1.42	.491	9.15	.010*	1.12	.571	5.81	.045*
**Current job**	28.4	.000*	1.89	.596	5.15	.161	.69	.876	1.68	.642
**Marital status**	8.93	.012*	2.72	.257	3.71	.156	3.66	.160	2.11	.349
**Nationality**	.68	.411	2.04	.153	3.46	.063	1.54	.215	8.57	.003*
**Place of residence**	9.94	.002*	5.90	.015*	.03	.856	1.93	.165	1.25	.263
**Family income**	2.56	.278	1.04	.594	2.21	.331	.53	.767	3.99	.136

*X*^*2*^ chi-square analysis

^*^Significant at < 0.05

## Discussion

This study was conducted to assess the level of awareness of the triage system among Jordanian patients who visit the ED. The current study found a low level of awareness about the triage system among patients receiving care in the ED in Jordanian public hospitals. This finding was consistent with studies performed in several countries [14, 21–23]. Addressing the low level of awareness necessitates the development of a culture that prioritizes learning and advancement to reinforce patient safety. Patients express willingness to receive more information about ED functions, as a measure that could enhance treatment quality and diminish wait times. This is in similar line with a study conducted by DeLaroche et al. [24] revealing that providing patients with thorough information about the ED’s triage system and procedures improves their overall experience and level of satisfaction. Therefore, improving patient education and communication presents a viable path to improving patient outcomes and streamlining emergency department healthcare delivery.

A positive correlation was found in Jordan’s healthcare system, indicating that a sizable number of patients were not utilizing primary care facilities efficiently which increases the country’s dependency on emergency rooms. This finding was consistent with a study conducted by AlShatarat et al. [14] that emphasizes the need for effective communication, documentation, and regular training in nursing care that highlights the need for healthcare professionals to be aware of these factors and tailor their care accordingly [25].

The study shows that patients’ understanding of the triage system varies; some are unfamiliar with the process of patient classification, while others are familiar with it. Nonetheless, the majority of respondents were now receiving care in an educational hospital and understood what an “educational hospital” provides services. The majority of patients were open to receiving updates regarding potential delays, and they preferred to hear them every 30 min. Similarly, a study was conducted by Alhabdan et al. [20] who found that the majority of patients are unaware of what triage is and are curious about how the emergency department operates. Furthermore, a large percentage of participants indicated that they lack a primary care provider. These findings are consistent with raising patient awareness through instruction and patient involvement in the event of a delay. A significant percentage of patients expressed interest in receiving further information about specific medical conditions and the healthcare system, indicating a desire for increased knowledge and understanding of their health and the care they receive.

The relevance of patient education and the possible influence of the healthcare setting on patient treatment and experience was highlighted by the belief held by more than half of the participants that teaching hospitals place a greater emphasis on patient education. Healthcare professionals need to understand the value of patient education and make sure patients have access to complete, accurate information about their illnesses and the healthcare system. By attending to patients’ needs for more information and comprehension, we enable them to take an active role in their care and make educated decisions about their health. Strategies to improve patient-centered education programs can be informed by an understanding of patients’ perceptions of teaching hospitals as having a stronger focus on patient education.

The study highlights that healthcare providers need to focus on enhancing patients’ understanding of the triage system to improve treatment quality and reduce wait times. Policymakers could incorporate guidelines and regulations to enhance the importance of effective communication and technology in triage processes. Future studies could focus more on improving triage awareness allocation and motivating patients to adopt a triage-aware culture.

### Implication

The current study has significant implications for healthcare practice, research, and policymakers. It is crucial to refine the triage concept to improve patient safety and ensure timely and appropriate delivery of medical care while taking into account available resources and survival probabilities. The study also highlights the need to enhance patients’ understanding of the triage system and the factors that influence their experiences during ED visits. Healthcare practitioners can use these findings to create interventions that increase patients’ awareness, improve treatment quality, and reduce wait times. Additionally, the study shows that patients’ access to timely and suitable medical care, considering available resources and survival prospects, significantly affects their understanding of the triage system and factors that shape their encounters during ED visits. Further research could explore ways to enhance triage awareness among patients and promote a culture of triage awareness. Nursing managers and policymakers should prioritize patient awareness of the triage system at EDs. This requires a strong commitment from healthcare institutions to prioritize patient triage system awareness as a core value, supported by investments in resources and training to strengthen these principles.

### Limitations

Several limitations should be acknowledged in our study. Firstly, our study was conducted in a single public hospital, which might constrain the applicability of the results to diverse healthcare settings or cultural contexts. Variances in healthcare systems and the implementation of triage protocols across different contexts may affect the study’s generalizability. Secondly, the utilization of self-reported data poses a potential limitation, given the susceptibility to social desirability bias, which might influence participants to present responses they believe are favorable to researchers or peers. Finally, the cross-sectional design of the study restricts the establishment of causal relationships between patients’ awareness of the triage system and their satisfaction with nursing care in the ED. To discern causality, longitudinal studies would be necessary.

## Conclusion

Most patients are not aware that there is a triage system. To improve patients’ understanding of the triage system, policymakers and stockholders ought to get in. More investigation is required to determine the causes of the low knowledge of the triage system. The study suggests increasing staffing levels, giving nursing staff ongoing education and training, integrating technology and automation to reduce the load of patient care, and fostering a supportive work atmosphere can all help patients become more conscious of the triage system in emergency departments. Subsequent investigations must concentrate on executing interventions such as task delegation, process re-engineering, and utilizing technology and automation. The efficacy of these approaches should be regularly assessed through feedback channels and evaluations.

## References

[1] Almarzooq AM (2020) Emergency department nurses’ knowledge regarding triage. Int J Nurs 7(2):29–44

[2] Eaid Elgazzar S (2021) Knowledge of triage and its correlated factors among emergency department nurses. Egypt J Health Care 12(4):1772–1780

[3] Zachariasse JM, Seiger N, Rood PP, Alves CF, Freitas P, Smit FJ, Moll HA (2017) Validity of the Manchester Triage System in emergency care: a prospective observational study. PloS one 12(2):e0170811 28151987 10.1371/journal.pone.0170811 PMC5289484

[4] Adeniji AA, Mash B (2016) Patients’ perceptions of the triage system in a primary healthcare facility, Cape Town, South Africa. Afr J Prim Health Care Family Medicine 8(1):1–9 10.4102/phcfm.v8i1.1148 PMC4926712 27380788

[5] Costa JPD, Nicolaidis R, Gonçalves AVF, Souza END, Blatt CR (2020) The accuracy of the Manchester Triage System in an emergency service. Rev Gaucha Enferm 41 10.1590/1983-1447.2020.20190327 33111760

[6] Napi N, Zaidan A, Zaidan B et al (2019) Medical emergency triage and patient prioritisation in a telemedicine environment: a systematic review. Heal Technol 9:679–700

[7] Cheng C, Zhang T, Su K et al (2019) Assessing the intensity of the population affected by a complex natural disaster using social media data. ISPRS Int J Geo Inf 8(8):358

[8] Townsend K, Loudoun R (2023) Line managers and extreme work: a case study of human resource management in the police service. Int J Hum Resour Manag 1–23

[9] Dolatabadi ZA, Zakerimoghadam M, Bahrampouri S, Jazini A, Foodani MN (2022) Challenges of implementing correct triage: a systematic review study. J Mil Med 24(2):1096–1105

[10] Bijani M, Khaleghi AA (2019) Challenges and barriers affecting the quality of triage in emergency departments: a qualitative study. Galen Med J 8:e1619 10.31661/gmj.v8i0.1619 PMC8344134 34466538

[11] FitzGerald G, Jelinek GA, Scott D, Gerdtz MF (2010) Emergency department triage revisited. Emerg Med J 27(2):86–92 20156855 10.1136/emj.2009.077081

[12] Joseph MJ, Summerscales M, Yogesan S et al (2023) The use of kiosks to improve triage efficiency in the emergency department. NPJ Digital Medicine 6(1):19 36737642 10.1038/s41746-023-00758-2 PMC9898217

[13] Tam HL, Chung SF, Lou CK (2018) A review of triage accuracy and future direction. BMC Emerg Med 18:1–7 30572841 10.1186/s12873-018-0215-0 PMC6302512

[14] AlShatarat M, Rayan A, Eshah NF et al (2022) Triage knowledge and practice and associated factors among emergency department nurses. SAGE Open Nursing 8:23779608221130588 36213615 10.1177/23779608221130588 PMC9536099

[15] Brevik HS, Hufthammer KO, Hernes ME et al (2022) Implementing a new emergency medical triage tool in one health region in Norway: some lessons learned. BMJ Open Quality 11(2):e001730 35534042 10.1136/bmjoq-2021-001730 PMC9086633

[16] Al-Kalaldeh M, Al-Bdour E, & Shosha GA (2021) Patients’ evaluation of the quality of emergency care services in Jordan: integration of patient centeredness model. Res Theory Nurs Pract 10.1891/RTNP-D-21-00037 34848564

[17] Kelen GD, Wolfe R, D’Onofrio G, Mills AM, Diercks D, Stern SA, Sokolove PE (2021) Emergency department crowding: the canary in the health care system. NEJM Catal Innov Care Deliv 2(5)

[18] Faul F, Erdfelder E, Lang A-G, Buchner A (2007) G* Power 3: a flexible statistical power analysis program for the social, behavioral, and biomedical sciences. Behav Res Methods 39(2):175–191 17695343 10.3758/bf03193146

[19] Seibert T, Veazey K, Leccese P, Druck J (2014) What do patients want? Survey of patient desires for education in an urban university hospital. West J Emerg Med 15(7):764 25493116 10.5811/westjem.2014.9.20674 PMC4251217

[20] Alhabdan N, Alhusain F, Alharbi A et al (2019) Exploring emergency department visits: factors influencing individuals’ decisions, knowledge of triage systems and waiting times, and experiences during visits to a tertiary hospital in Saudi Arabia. Int J Emerg Med 12(1):1–8 31752662 10.1186/s12245-019-0254-7 PMC6868738

[21] Alharbi NS, Youssef HA, Felemban EM, Alqarni SS, Alharbi NM, Alsayed AAO, Shahbal S (2022) Saudi emergency nurses preparedness for biological disaster management at the governmental hospitals. J Posit School Psychol 6(9):1218–1235

[22] Mirmozaffari M, Kamal N (2023) The application of data envelopment analysis to emergency departments and management of emergency conditions: a narrative review. Paper presented Healthcare 10.3390/healthcare11182541 PMC10530342 37761738

[23] Zachariasse JM, van der Hagen V, Seiger N, Mackway-Jones K, van Veen M, Moll HA (2019) Performance of triage systems in emergency care: a systematic review and meta-analysis. BMJ open 9(5) 10.1136/bmjopen-2018-026471 PMC6549628 31142524

[24] DeLaroche, AM, Rodean, J, Aronson PL, Fleegler, EW, Florin TA, Goyal M, Sills, MR (2021) Pediatric emergency department visits at US children’s hospitals during the COVID-19 pandemic. Pediatrics 147(4) 10.1542/peds.2020-039628 33361360

[25] Malak MZ, Al-Faqeer NM, Yehia DB (2022) Knowledge, skills, and practices of triage among emergency nurses in Jordan. Int Emerg Nurs 65:101219 36323189 10.1016/j.ienj.2022.101219

